# Needs for discharge planning among parents of preterm infants in the NICU: a systematic review and meta-synthesis

**DOI:** 10.3389/fpubh.2025.1667721

**Published:** 2025-11-06

**Authors:** Jiaming Wu, Longli Li, Xin Guo, Xue Hu

**Affiliations:** 1 Xing Yi People’s Hospital, Xingyi, China; 2 Yunnan University of Traditional Chinese Medicine, Kunming, China; 3 Yan'an Hospital Affiliated to Kunming Medical University, Kunming, China

**Keywords:** infant premature, parents, needs, qualitative research, neonatal intensive care units, post-discharge support

## Abstract

**Objectives:**

To identify the discharge preparation service needs of parents of preterm infants through a qualitative systematic review and meta-synthesis.

**Methods:**

A systematic search was conducted for qualitative studies examining the discharge preparation needs, experiences, and perceptions of parents of preterm infants. The search was limited to publications in English and Chinese, as these were the languages in which the research team was proficient, ensuring accurate comprehension and interpretation of the nuanced qualitative data. The inclusion criteria were as follows: (P) Parents of preterm infants hospitalized in the NICU or parents of preterm infants discharged from the NICU; (I) Needs, experiences, and difficulties encountered by parents of preterm infants in preparation for hospital discharge; (Co) Follow-up of preterm infants during hospitalization in the NICU or in the weeks following discharge from the NICU; (S) Various types of qualitative research. Two independent reviewers screened titles and abstracts, assessed the full texts of potentially eligible studies for inclusion, evaluated the methodological quality of the included studies, and extracted the data. Discrepancies were resolved through discussion or consultation with a third reviewer.

**Results:**

A total of 12 studies revealed 3 descriptive themes and 9 sub-themes: (1) psychological and social support needs, (2) information and skills support needs, (3) continuity of services and resource requirements.

**Conclusion:**

During the preparation for discharge of parents of preterm infants in the NICU, their needs for psychological support, knowledge, and skills in preterm infant care, as well as post-discharge support, are evident. Healthcare professionals should address their psychological needs, facilitate family role adaptation, provide individualized health education, and strengthen the post-discharge support system to enhance parents’ ability to care for preterm infants at home.

**Systematic review registration:**

https://www.crd.york.ac.uk/PROSPERO/.

## 1 Introduction

According to a report by the World Health Organization (WHO), the global incidence of preterm birth ranges from 5.0 to 18.0% and demonstrates an increasing trend annually (1). China’s preterm birth rate is approximately 6.7 to 7.3%, ranking second globally (2). Due to factors such as immature organ and system development and compromised immune function, preterm infants are susceptible to multiple complications, making preterm birth one of the leading causes of mortality among children under 5 years of age worldwide (3). These infants typically require transfer to a neonatal intensive care unit (NICU) for treatment and observation after birth. They exhibit poor environmental adaptability (4), and post-discharge care presents significant challenges. Common risks include feeding difficulties, apnea, and infections, resulting in elevated readmission rates (5). Research indicates that over 30.0% of preterm infants may be readmitted within 3 months of discharge due to related health issues (6). Currently, influenced by China’s NICU management model and prolonged hospital stays, parents of preterm infants often lack adequate discharge preparation, making it difficult to meet their children’s post-discharge care needs (7).

Discharge planning services (also referred to as discharge planning) represent a patient-centered, needs-driven model. Through active participation and collaboration among patients, their families, and a multidisciplinary team across multiple institutions, this approach serves as the predominant practice model for ensuring continuity of care. This methodology primarily targets patient populations with complex post-discharge care requirements or those at risk of delayed discharge (8). The Canadian Paediatric Society emphasizes that discharge readiness for preterm infants encompasses not only clinical recovery but also family preparedness. Inadequate family readiness, coupled with caregivers’ insufficient knowledge and skills, may hinder the transition to home care. Such barriers can impede growth, development, and rehabilitation while increasing the risk of complications leading to readmission (9). Internationally, well-established systems for discharge preparation services have been developed (10, 11), whereas related practices in China’s NICUs remain in the preliminary exploration phase (12). Consequently, obtaining a comprehensive understanding of the genuine needs and perceptions of parents of premature infants regarding discharge planning holds significant practical importance. Although numerous studies in recent years have examined the discharge planning needs of parents of preterm infants, existing knowledge remains fragmented due to variations in national and institutional contexts, making it challenging to fully capture the authentic experiences of this population.

## 2 Methods

### 2.1 Objectives

This study employed a systematic review and meta-synthesis methodology to synthesize published qualitative research on the needs of parents of preterm infants regarding discharge preparation services. The objective was to elucidate these parents’ needs and experiences during the discharge preparation process, with the ultimate goal of providing evidence-based support for developing tailored discharge preparation service programs within the Chinese context.

### 2.2 Design

This qualitative systematic review and meta-analysis was conducted and reported in accordance with an established methodological framework to ensure rigor and transparency. The research methodology followed the Joanna Briggs Institute (JBI) Evidence Synthesis Handbook (13), which provides comprehensive guidance for all stages of qualitative systematic reviews, including question formulation, search strategies, study selection, quality assessment, and data synthesis. Additionally, the study adheres to the Enhancing Transparency in Reporting the Synthesis of Qualitative Research (ENTREQ) Statement (14), which consists of a 21-item checklist designed to improve the clarity and completeness of reporting in qualitative evidence synthesis. The study selection process is presented using a flow diagram recommended by the Preferred Reporting Items for Systematic Reviews and Meta-Analyses (PRISMA) statement, clearly illustrating the identification and screening of studies. This meta-synthesis protocol is registered with PROSPERO (ID: CRD42024519484).

### 2.3 Inclusion and exclusion criteria

#### 2.3.1 Inclusion criteria

The inclusion criteria were established based on the PICoS framework:

- Participant (P): Parents of preterm infants hospitalized in the NICU or parents of preterm infants discharged from the NICU.
- Phenomena of interest (I): Needs, experiences, and difficulties encountered by parents of preterm infants in preparation for hospital discharge.
- Context (Co): Follow-up of preterm infants during hospitalization in the NICU or in the weeks following discharge from the NICU.
- Study design (S): Various forms of qualitative research were eligible, including, but not limited to, phenomenological, descriptive qualitative, and narrative studies.

#### 2.3.2 Exclusion criteria

The exclusion criteria for the studies are as follows:

- Duplicate reports: Reports presenting identical results from the same cohort (only the most comprehensive or earliest report was retained).
- Inaccessible data: Studies for which full-text access could not be obtained despite exhaustive efforts (e.g., through interlibrary loan or direct contact with the authors).
- Inappropriate study design: Non-qualitative research, including but not limited to quantitative studies, systematic reviews, meta-analyses, meta-syntheses, research protocols, and commentary articles. Additionally, studies that did not constitute primary qualitative research were excluded.
- Publication type: Conference abstracts, theses, and dissertations, which could not be effectively extracted or synthesized due to insufficient qualitative data and analysis.
- Language restrictions: Studies published in languages other than English or Chinese were excluded due to practical limitations in translation and interpretation.

### 2.4 Search strategies

Nine databases, including PubMed, the Cochrane Library, Web of Science, Embase, MEDLINE, China National Knowledge Infrastructure (CNKI), VIP Chinese Science and Technology Periodical Database (VIP), China Biology Medicine disc (CBM), and Wanfang Database (WANFANG), were systematically searched. The search period spanned from the inception of each database to April 17, 2025. The search strategy employed both Medical Subject Headings (MeSH) and free-textterms, including: infant newborn, newborn*, neonate*, premature, infant premature, infant, premature newborn, parents, parent*, father*, mother*, maternal, paternal, discharge planning, discharge preparation, discharge preparation service, plan for discharge, discharge readiness, qualitative research, descriptive analysis*, interview, content analysis*, thematic analysis*, grounded theory, phenomenology, and qualitative study. To ensure a comprehensive literature search, Boolean operators were used to combine search terms, and manual searches were conducted. The complete search strategies for all nine databases are provided in Supplementary file 1.

### 2.5 Study selection and data extraction

The study screening process adhered to the PRISMA guidelines. Following the literature search, all retrieved records were imported into NoteExpress 3.7 for duplicate removal. Two reviewers (WJM and LLL) independently conducted the subsequent screening in two phases. First, both reviewers independently evaluated the titles and abstracts of all retrieved records based on predefined inclusion and exclusion criteria. Subsequently, full texts of potentially eligible studies were obtained and independently assessed for eligibility by the same reviewers. Discrepancies during both phases were resolved through discussion, with consultation of a third reviewer when necessary to achieve consensus.

After final inclusion decisions, the same reviewers (WJM and LLL) independently extracted data using the JBI standardized data extraction tool for qualitative research designs (13). The extracted data included authors, publication year, country, study design, study population and sample size, phenomenon of interest, and key findings pertinent to the review question. The extracted data were cross-verified, with inconsistencies resolved through discussion or third-party reviewer intervention. The remaining researchers validated the final extracted data to ensure accuracy.

### 2.6 Quality appraisal

The included literature was independently evaluated by two researchers (WJM and LLL) using the JBI Center for Evidence-Based Healthcare’s quality assessment criteria for qualitative research (15). In cases of disagreement, the decision was discussed with a third researcher. The remaining researchers then reviewed the quality assessment results to ensure reliability. The assessment criteria examined 10 domains, including the appropriateness of research methods, clarity of research questions, data analysis procedures, consistency between interpretations and findings, cultural or theoretical alignment with the researcher’s presentation, researcher influence on the study, adequate representation of participant perspectives, ethical approvals, and logical conclusions drawn from the data. Each item was scored 1 point if supported by evidence in the study and 0 points otherwise, yielding a total score ranging from 0 to 10 (16). The research team discussed and adopted the following quality classification: high (8–10 points), moderate (4–7 points), and low (0–3 points) (17).

### 2.7 Data synthesis

Data synthesis employed inductive thematic analysis following the meta-synthesis methodology recommended by the JBI (13). This approach aims to derive novel insights from the raw findings of qualitative research. The specific procedure was as follows: Two primary researchers (WJM and LLL) independently and repeatedly reviewed the findings of each included study to achieve deep immersion and comprehensive familiarity. Meaningful concepts were extracted from the research findings and assigned descriptive codes. Subsequently, codes were grouped based on similarity and conceptual coherence (i.e., sharing a common core idea). Finally, comprehensive descriptive themes were developed through abstract synthesis to summarize the core experiences and needs of the parent group.

It is particularly important to note that in qualitative meta-synthesis research—especially when integrating complex human experiences—findings from individual primary studies may simultaneously support multiple themes. Consequently, the resulting themes are not strictly mutually exclusive. A single participant quotation or finding may reflect dimensions of multiple themes, demonstrating the interconnected nature of parental experiences (e.g., information needs are closely related to anxiety reduction). This synthesis aims to construct a coherent and nuanced representation of the data, prioritizing accurate depiction of the research phenomena over rigid, mutually exclusive categorization. Details of the meta-synthesis results are provided in Supporting file 2.

## 3 Results

### 3.1 Procedure for extraction

**Figure 1:**
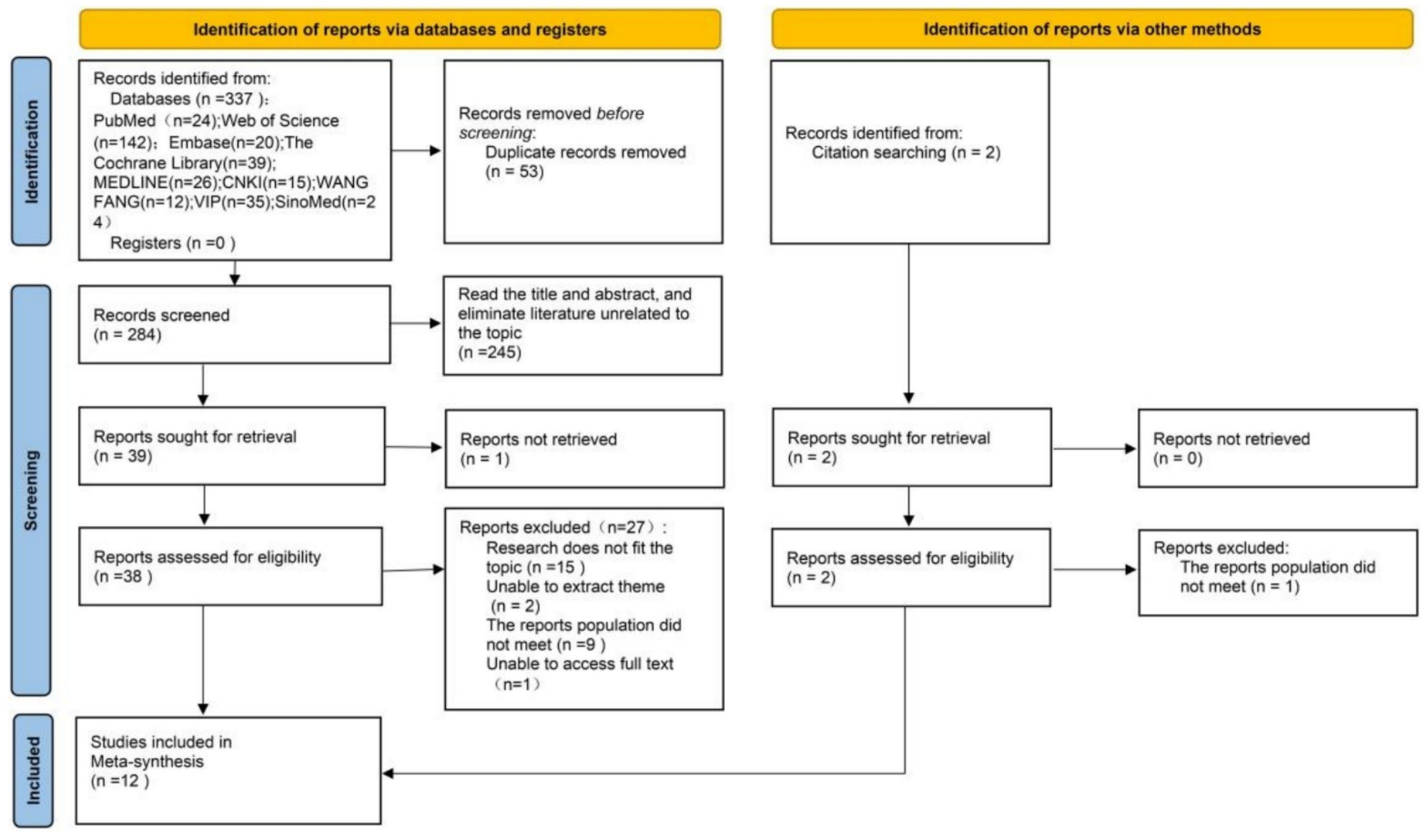
PRISMA flowchart.

The initial search identified 337 reports. After removing duplicates, 284 reports remained. Screening titles and abstracts and excluding reports irrelevant to the topic resulted in 39 reports. Of these 39 reports assessed in full text, 11 met the inclusion criteria. One additional study was identified through reference list and citation searches, yielding a final total of 12 included studies. Reasons for exclusion were as follows: reports outside the scope of the topic, inability to extract relevant themes, report populations not meeting eligibility criteria, and unavailability of full-text access. Two researchers (WJM and LLL) independently conducted each stage of the literature selection process. Results were compared and discussed to resolve any discrepancies. The study selection process is illustrated in Figure 1.

### 3.2 Basic characteristics of the included literature

A total of 12 studies met the inclusion criteria, involving 204 parents of preterm infants from China, the United States, Canada, Indonesia, and Kenya. Various methodologies were employed, including nine phenomenological studies, two descriptive qualitative studies, and one narrative interview (Table 1).

**Table 1:** Key characteristics and quality appraisal of 12 included studies.

Authors, Year	Country	Research method	Sample size	Interest of phenomena	Results
Sun et al. (2023) (18)	China	Phenomenological approach	15	An in-depth look at the real-life experiences of fathers of preterm infants in the NICU who are prepared for discharge from the hospital	Four themes: Preterm fathers’ distress and growth; lack of independent caregiving; family-society role conflict; desire for support
Yu et al. (2024) (19)	China	Phenomenological approach	12	Exploring parents’ readiness for hospital discharge and home care information needs	Four themes: Diverse knowledge needs; inadequate role adaptation; uncertain discharge readiness; emotional changes
Jiang and Jiang (2022) (20)	China	Phenomenological approach	15	Understanding the needs of parents of preterm infants discharged from the NICU	Four themes: Caregiving knowledge and skills needs; illness protection needs; outside support needs; autonomous caregiving needs
Zhou et al. (2011) (21)	China	Phenomenological approach	18	Understanding the Breastfeeding Experiences of Mothers of Preterm Infants Discharged from the NICU	Four themes: Difficulties in direct breastfeeding; confusion about uncertainty of breastfeeding; worries about insufficient breast milk; lack of knowledge about breastfeeding
Yue et al. (2021) (22)	China	Phenomenological approach	12	Understanding the Health Education Needs of Mothers of Very Preterm Infants in the NICU	Three themes: Lack of basic parenting knowledge and skills; changes in mothers’ focus in parenting as the duration of parenting increases; urgent need for various forms of pre-discharge health education
Zhou et al. (2012) (23)	China	Phenomenological approach	16	Understanding mothers’ experiences of parenting their preterm babies in the first month after discharge from the hospital	Five theme: Confusion over breastfeeding difficulties and uncertainty; feelings of overwhelm over low parenting skills; uneasiness over common symptoms of preterm babies; feelings of parenting burden; confusion and uneasiness due to parenting information
Zhang et al. (2020) (24)	China	Phenomenological approach	18	Understanding mothers’ perceptions and experiences of breastfeeding after discharge of very low birth weight infants from the hospital	Four themes: Lack of knowledge about breastfeeding; difficulties in breastfeeding; willingness to breastfeed; need for breastfeeding support
Reyna et al. (2006) (25)	USA	Descriptive qualitative analyses	27	Exploring mothers’ perceptions of their experiences of feeding their preterm infants in the first weeks after hospital discharge	Three themes: Explaining infant behavior; managing the evolving feeding process; recognizing knowledge gaps
Premji et al. (2017) (26)	Canadian	Phenomenological approach	11	Exploring mothers’ experiences of caring for late preterm infants in the community	Four themes: Caring for late preterm infants; feeding late preterm infants; experiences of public health nurses; mood and emotional disorders
Hariati et al. (2021) (27)	Indonesia	Descriptive qualitative analyses	8	Exploring preterm mothers’ experiences of home care one month after their babies are discharged from the hospital	Three themes: Transition to independent motherhood; focus on care of infants after discharge from the hospital; barriers and facilitators to care
Maluni et al. (2025) (28)	Kenya	Narrative interviews	34	Exploring the experiences of mothers of preterm babies after discharge from the hospital	Three themes: Feelings during discharge: excitement and fear; information provided and needed at discharge; experiences and challenges once home
Jiang et al. (2024) (29)	China	Phenomenological approach	18	Explore the breastfeeding experiences of mothers of preterm infants and the challenges that affect their breastfeeding practices	Four themes: Breastfeeding motivation; breastfeeding challenge; breastfeeding support and education; response to parental stress

### 3.3 Quality appraisal results

The methodological quality of the 12 included studies was assessed using the JBI qualitative research appraisal tool, which evaluates 10 dimensions, including the appropriateness of the study design, adherence to ethical standards, and the rigor of data collection and analysis. The results indicated that 7 studies (58.3%) were rated as high quality, demonstrating clear objectives, transparent methodologies, and sufficient data analysis. The remaining 5 studies (41.7%) were rated as moderate quality, with certain limitations, such as unclear alignment between philosophical underpinnings and methodology, as well as insufficient description of the researcher’s influence on the study or the study population’s impact on the research (Table 2).

**Table 2:** Results of the methodological quality assessment of the included literature.

Authors, Year	①	②	③	④	⑤	⑥	⑦	⑧	⑨	⑩	Overall evaluation
Sun et al. (2023) (18)	Unclear	Yes	Yes	Yes	Yes	No	No	Yes	Yes	Yes	Moderate
Yu et al. (2024) (19)	Unclear	Yes	Yes	Yes	Yes	No	No	Yes	Yes	Yes	Moderate
Jiang and Jiang (2022) (20)	Unclear	Yes	Yes	Yes	Yes	Yes	No	Yes	Yes	Yes	High
Zhou et al. (2011) (21)	Unclear	Yes	Yes	Yes	Yes	No	No	Yes	Yes	Yes	Moderate
Yue and Zhang (2021) (22)	Unclear	Yes	Yes	Yes	Yes	No	No	Yes	Yes	Yes	Moderate
Zhou et al. (2012) (23)	Yes	Yes	Yes	Yes	Yes	No	Yes	Yes	Yes	Yes	High
Zhang et al. (2020) (24)	Unclear	Yes	Yes	Yes	Yes	No	No	Yes	Yes	Yes	Moderate
Reyna et al. (2006) (25)	Yes	Yes	Yes	Yes	Yes	No	No	Yes	Yes	Yes	High
Premji et al. (2017) (26)	Yes	Yes	Yes	Yes	Yes	Yes	No	Yes	Yes	Yes	High
Hariati et al. (2021) (27)	Yes	Yes	Yes	Yes	Yes	Yes	No	Yes	Yes	Yes	High
Maluni et al. (2025) (28)	Yes	Yes	Yes	Yes	Yes	Yes	No	Yes	Yes	Yes	High
Jiang et al. (2024) (29)	Yes	Yes	Yes	Yes	Yes	Yes	No	Yes	Yes	Yes	High

① Is there congruity between the stated philosophical perspective and the research methodology? ② Is there congruity between the research methodology and the research question or objective? ③ Is there congruity between the research methodology and the methods used to collect data? ④ Is there congruity between the research methodology and the representation and analysis of data? ⑤ Is there congruity between the research methodology and the interpretation of results? ⑥ Is there a statement locating the researcher culturally or theoretically? ⑦ Is the influence of the researcher on the research, and vice versa, addressed? ⑧ Are participants, and their voices, adequately represented? ⑨ Is the research ethical according to current criteria or, for recent studies, is there evidence of ethical approval by an appropriate body? ⑩ Do the conclusions drawn in the research report flow from the analysis or interpretation of the data?

### 3.4 Themes

**Figure 2:**
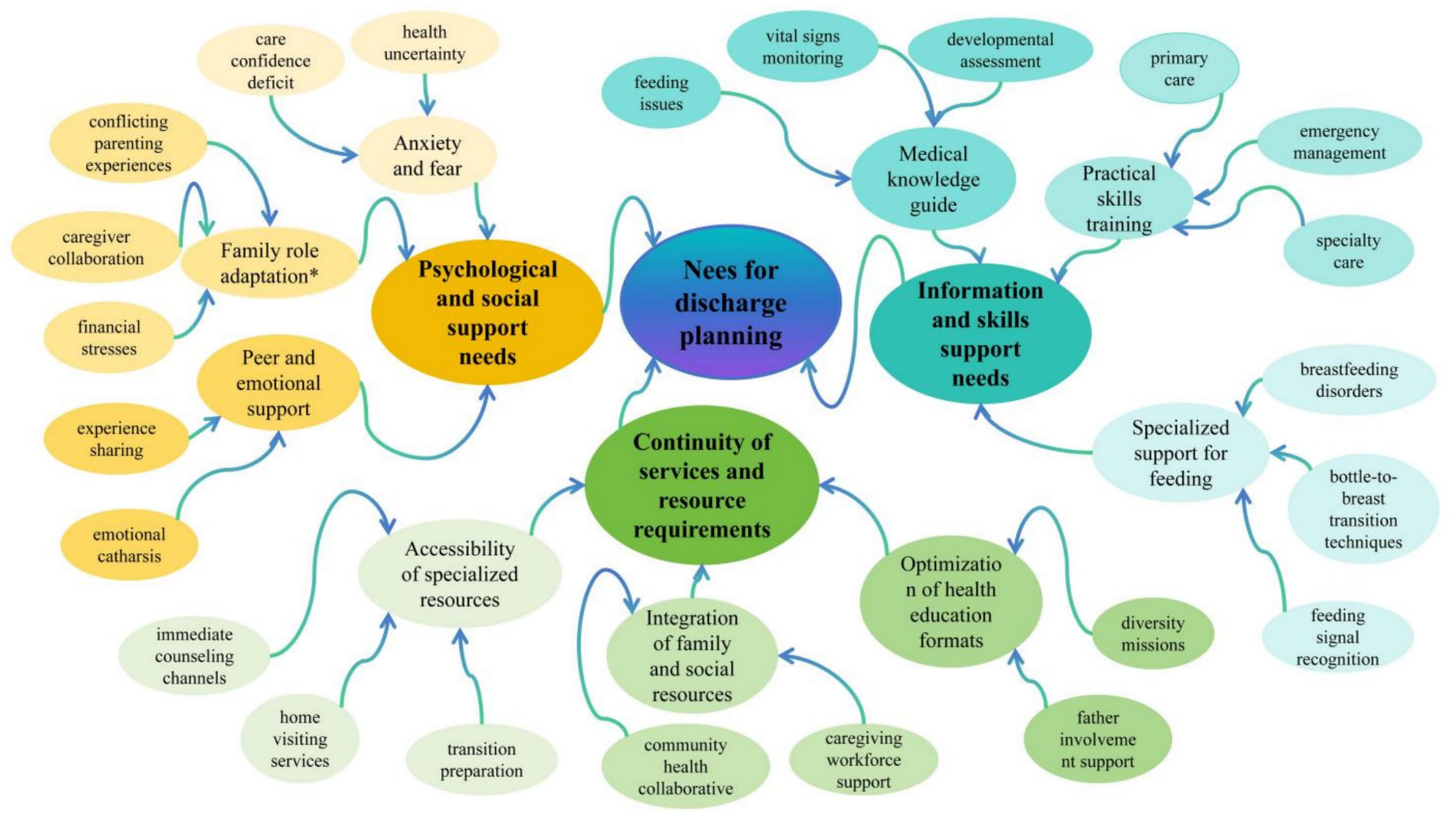
PRISMA integration results relationship map. *Family role adaptation: Following the discharge of a premature infant, both parents undergo a process of readjustment and redefinition of their respective roles, behavioral patterns, and mutual relationships within the family to address new parenting challenges.

The two researchers (WJM and LLL) systematically reviewed, analyzed, and compared 12 studies, identifying a total of 23 findings. Similar findings were categorized into nine subthemes and synthesized into three descriptive themes (Figure 2).

#### 3.4.1 Meta-theme 1: psychological and social support needs

##### 3.4.1.1 Sub-theme 1: anxiety and fear

For parents of preterm infants in the NICU, intense health uncertainty and a profound lack of confidence in caregiving represent major sources of anxiety and fear as they prepare for their child’s discharge from the hospital. First, health uncertainty persists as a significant concern (19, 22, 23, 27). Even when discharge criteria are met, the fragile vital signs of preterm infants, potential risk of complications, and unpredictable long-term developmental outcomes contribute to parental anxiety regarding future outcomes. Parents express concern about whether their child will remain stable after transitioning from specialized care. This prognostic uncertainty perpetuates deep-seated fears.

Second, a substantial lack of confidence in caregiving exacerbates this anxiety (18, 22, 23, 28), as parents often feel inadequately prepared to manage a preterm infant requiring specialized care. Even with prior parenting experience, they may lack the necessary knowledge and skills for delicate feeding, vital sign monitoring, recognizing abnormalities, managing oxygen-dependent discharge, or providing rehabilitation stimulation for fragile preterm infants. This self-doubt regarding their caregiving competence manifests as fear of independent care—specifically, concerns that their child’s health may be compromised due to parental oversight or error—along with feelings of helplessness in emergency situations.

The intersection of profound concern about their child’s uncertain health outcomes and limited confidence in their own caregiving abilities constitutes the primary source of anxiety and fear for NICU parents preparing for discharge. For example:

*“When I was discharged from the hospital, I thought it would be good to come back, but I did not realize that it would be totally different after I came back! My baby was not feeding well and was often bloated, so I was very anxious and felt that it was better to stay in the hospital”* (18).

*“I’m wondering if she’ll fall behind other kids her age and if there are any other implications?”* (19).

*“I did not dare to hold her for the first two days after she came back, thinking she was too small and soft. I did not dare to walk after picking her up for fear that she would fall”* (22).

*“I did not know how to do anything; it was my grandmother who did it, and I did not dare to take him out of the hospital without her help. One day when Grandma went out, the baby’s poop got everywhere, and I was so anxious that I cried along with him, calling my mom to tell her to come back soon”* (23).

*“I keep checking her breathing because I’m afraid she’s going to stop”* (27).

*“You do not know what you are feeling at that point. It was the… okay, the thing is, it comes with fear, you are excited, you really want to go home, but this child is still tiny, because their discharge weight is 1.75 (Kg) up to 2kgs depending on if the baby is feeling good. So, if the baby is stable… mine was born at 1700 grams. What are you going to do with this baby? You know, here you were confident, if he vomits a little, “doctor the baby is vomiting,” he tells you, “aahh… mum bend the baby and do this.” Now you have been told to go home, you will be alone, whatwill happen? “* (28).

##### 3.4.1.2 Sub-theme 2: family role adaptation

Following hospital discharge, parents of preterm infants face substantial challenges in role reconstruction. First, discrepancies frequently arise between parents and elders regarding specialized care concepts and methods for fragile preterm infants (22). The absence of standardized care protocols leads to disputes and recurrent conflicts over parenting practices, thereby exacerbating parental anxiety.

Second, parents often experience an inequitable division of labor and communication breakdowns due to work demands, emotional stress, or differences in caregiving competencies (18, 19, 27, 29). Typically, one parent (most often the mother) assumes an excessive caregiving burden, resulting in physical and psychological exhaustion, while the other parent may experience frustration and detachment due to an inability to contribute meaningfully or provide sufficient support. The lack of structured caregiver collaboration further undermines the foundation for cooperative coping.

Finally, persistent financial pressures impose a significant burden on parental role adaptation (19). High medical expenses, specialized formula costs, rehabilitation fees, and potential reductions in household income compel parents to endure considerable stress in financial decision-making, which may trigger conflicts regarding resource allocation and career adjustments. In some cases, this financial strain necessitates extended work hours for one parent (typically the father) to maintain household solvency, further restricting their availability for childcare and family collaboration, thereby perpetuating a detrimental cycle. For example:

*“I’ve been studying lately… I hope I can be a competent husband and father”* (18).

*“… I work during the day and take care of them at night after work”* (19).

*“He lives inside, we live outside. It costs money. It’s a big burden”* (19).

*“It is said that the eldest is raised according to books and the youngest according to pigs. In our case, the oldest was raised according to books, and the second had to be raised according to books even more. Because she was born prematurely, I feel that she is completely different from the oldest when she was a baby, so I cannot raise her with the same experience as the oldest. I feel like I’m learning all over again!”* (22).

*“I was particularly challenged in taking care of my infant. It was almost the same as infants in general. But I had to undertake more intensive monitoring of my infant”* (27).

*“I am the father of a baby, and I am always criticized by my family for being clumsy and not being allowed to touch the baby. I want to take care of my child, and I hope my family recognizes and supports me”* (29).

##### 3.4.1.3 Sub-theme 3: peer and emotional support

The lack of experience-sharing channels presents challenges for parents of preterm infants in obtaining targeted guidance (24, 29). Conventional parenting methods often prove inadequate for the specialized care required by fragile preterm infants (e.g., feeding difficulties, respiratory monitoring, and interventions for developmental delays). These parents seek to exchange practical advice, coping strategies, and resource information with others who have faced similar circumstances to alleviate the substantial anxiety caused by uncertainty. They also aim to prevent recurring challenges resulting from isolation and insufficient information.

Additionally, parents of preterm infants experience unresolved concerns regarding their infants’ health, frustration in the caregiving process, and complex emotions (e.g., guilt, anger, sadness) that others may struggle to comprehend. However, they frequently suppress these feelings due to fear of judgment or imposing burdens on others. Their opportunities for emotional release are severely restricted (24, 29), lacking both a safe space to confide in peers and access to professional emotional counseling. These unaddressed negative emotions accumulate, exacerbating parental psychological distress (e.g., increased risk of depression) and impairing caregiving capacity and family dynamics, perpetuating a harmful emotional cycle. Their heightened sensitivity to negative evaluations further discourages them from seeking external support, often leaving them in profound emotional isolation. Consequently, they urgently require effective peer and emotional support. For example:

*“I think it’s very good that the hospital has set up a group for mothers of preterm babies. For example, some babies will have hemolysis after birth, and to a certain extent, they will need to have their blood replaced. Mothers who do not know the situation will be scared to death when they hear the term “blood exchange,” but it’s different when we communicate with each other in the group. I feel much more relieved when I learn from other people’s experiences, and I usually consult with the group when I have questions about breastfeeding. Everyone is very willing to help!”* (24).

*“I used to cry in secret when my family wasn’t around. My lover and friends have been doing the thinking for me, and I feel better now”* (24).

*“After the baby came home, the color of the stool got lighter and lighter. Suspecting it was biliary atresia, I became very depressed. I did not dare to talk to the older people at home, afraid they would feel the pressure. Sometimes I would talk to my friends, and they would comfort me, which made the pressure feel less”* (29).

*“We would burp our babies after feeding, but we could never burp them well, so we joined a couple of NICU preemie family parents’ WeChat groups. Experienced parents in the group taught us. It’s good to be in this group. When we feel bad, parents who have had similar experiences share their own stories with us to relieve our stress and help us get through this difficult time!”* (29).

#### 3.4.2 Meta-theme 2: information and skills support needs

##### 3.4.2.1 Sub-theme 4: medical knowledge guide

Parents of fragile preterm infants require precise and actionable medical guidance following hospital discharge to address their primary concerns and enhance caregiving confidence. Due to preterm infants’ underdeveloped sucking ability, limited gastric capacity, and susceptibility to regurgitation or feeding refusal, parents often lack clear instructions regarding feeding volume, frequency, positioning (particularly during transitions between breastmilk and specialized formulas), and emergency responses to choking (19, 21, 23, 24, 29). This uncertainty contributes to apprehension during feeding, exacerbated sleep deprivation due to frequent nighttime feedings, and self-blame and anxiety stemming from feeding difficulties and associated psychological stress.

Additionally, parents lack sufficient knowledge to monitor preterm infants’ vital signs (20–22), making it difficult to accurately identify critical indicators such as apnea, cyanosis, abnormal body temperature, or signs of infection. They may respond with excessive anxiety to minor infant changes or delays in addressing them, resulting in persistent distress and a heightened sense of helplessness regarding perceived instability.

Furthermore, the absence of standardized developmental assessment criteria intensifies parental concerns (19, 22, 28). Parents urgently need information on appropriate developmental milestones for preterm infants at corrected age, early intervention indicators, and long-term prognosis. However, the inapplicability of typical parenting benchmarks heightens their focus on their infants’ developmental progress. Any indication of delay may trigger profound concern regarding growth, development, and prognosis, or even unnecessary self-doubt or external pressure due to misinterpretation. Without this essential medical knowledge, parental fears and anxieties remain unresolved, significantly increasing the caregiving burden. For example:

*“Baby’s stools often have some white stuff mixed in… Is it a digestive problem?”* (19).

*“Because it is a premature baby… It may have growth retardation after discharge from the hospital”* (19).

*“The doctor said to monitor the baby’s temperature after discharge. How should I measure it? Which part of the body should be measured? For how long?”* (20).

*“I heard that rice and soybean paste are highly nutritious, but I was worried about the baby’s indigestion, so I did not dare to feed it. Now I’m really scared to think about it—if it causes indigestion, I’ll have to be hospitalized again”* (21).

*“Why is my child breathing so fast—much faster than me? Is he having trouble breathing? The night I was discharged from the hospital, I took him back because it seemed like he was breathing quickly. Although the doctor said he was fine, I still felt uneasy”* (21).

*“I’ve been worried about my baby’s belly button rising 2 centimeters when he cries”* (22).

*“I’m worried about his development. Look at me now; I always think he’s short”* (22).

*“The baby spits up a lot; twice it came out of his nose, and the family had to take turns sleeping at night. How scary if the spitting up goes unnoticed!”* (23).

*“When the baby was first discharged from the hospital, she cried very hard and would not breastfeed. I thought she was sick, but the doctor checked her and said she was fine. I think I’m too sensitive and get very nervous when she does not want to breastfeed, and I feel really tired of this!”* (24).

*“Like I have a neighbor—they are one month apart from my son. But you know, my son cannot walk on his own, but that kid can walk now… Yeah. So, someone goes, ‘Ah, when was your kid born?’ ‘You know, um, has he not started walking…”* (28).

*“My baby was born prematurely and admitted to the NICU. Although I was told to express or pump breastmilk during the hospitalization, I still did not know how to breastfeed after my baby was discharged”* (29).

##### 3.4.2.2 Sub-theme 5: practical skills training

With the imminent discharge of preterm infants from hospitals, the urgent need for parents to master essential care skills significantly exceeds that of parents of full-term newborns. This necessity is directly linked to their primary concerns regarding “helplessness in abnormal situations” and “the unstable condition of preterm infants.” First, inadequate foundational care skills exacerbate daily stress (20, 22, 23). Parents must repeatedly practice basic procedures—such as safe holding, umbilical cord disinfection, skin care (particularly to prevent damage to the fragile skin of preterm infants), and bathing and maintenance—under professional supervision. A lack of proficiency in these tasks results in rigid movements and prolonged, inefficient procedures, intensifying the already exhausting demands of daily care and worsening parental fatigue and sleep deprivation.

Second, the inability to manage emergencies—such as choking, asphyxia, apnea, fever, and convulsions—heightens parental distress (22, 26). Parents express a strong desire for simulation training to master correct back patting techniques, infant cardiopulmonary resuscitation (CPR), and emergency response protocols. The absence of these skills, coupled with persistent “fear and anxiety” from constant vigilance, fuels concerns about delayed intervention and treatment.

Finally, specialized care requirements—including gastrostomy tube feeding, oxygen therapy monitoring, ostomy care, or infant rehabilitation—present unique challenges (22, 23, 27, 28). Parents must precisely master aseptic techniques, equipment operation, and the identification of abnormalities (e.g., signs of infection). Any error may compromise infant safety or precipitate complications, further amplifying concerns about “development and prognosis.” Without these core competencies, parents’ feelings of helplessness and anxiety remain unmitigated, and confidence in caregiving remains difficult to establish. For example:

*“There are so many straps on the baby’s clothes, I really do not know which one to tie to which one, and the bag I wrapped myself in was loosened by the baby’s kick!”* (20).

*“When I was first discharged from the hospital, I did not know how to hold him, I did not know how to wrap him, and I did not know how to burp him because his head kept swaying back and forth when he sat up”* (22).

*“When I first came home from the hospital, I was worried that he had stopped breathing. He stopped breathing while breastfeeding; he could not breathe enough and turned purple. I was very worried”* (22).

*“The baby’s mouth is so small, I do not dare to open it… I’m really at a loss when it comes to giving medicine to my baby”* (22).

*“When will the jaundice on the baby’s face go away completely? Is there a problem? Should I go to the hospital?”* (23).

*“Sometimes babies sleep for 6–7 h without waking up, and I want to wake them to feed them, but I cannot get them to wake up. I’m confused”* (23).

*“He was hypothermic, so I was a little nervous and upset… So of course I got frustrated and started crying”* (26).

*“I did KMC for three days after returning home from the hospital, but I did not it anymore because my infant was healthy. KMC was difficult and I needed to be helped by others as I cannot do it alone “* (27).

*“Yeah, they explained to us how to handle the baby well at home, especially insisting that we continue with kangaroo mother care… Until they reach those kilograms. Kangaroo mother care helps the baby gain weight fast…”* (28).

##### 3.4.2.3 Sub-theme 6: specialized support for feeding

Feeding sessions represent one of the primary stressors encountered by parents of preterm infants following hospital discharge, highlighting the need for specialized and individualized support. Breastfeeding difficulties present significant physical and psychological challenges. Preterm infants exhibit underdeveloped sucking-swallowing-breathing coordination and are prone to fatigue, frequently resulting in breastfeeding complications, inefficient feeding, or maternal nipple trauma. Mothers experience tension between their strong motivation to breastfeed (particularly due to the perceived immunological benefits) and the practical challenges they face (e.g., inadequate milk production, infant refusal of breastfeeding) (21, 24, 28). Repeated unsuccessful attempts often lead to profound frustration, guilt, and self-doubt, which exacerbate feeding-related anxiety and stress. The necessity for frequent milk expression and nighttime breastfeeding to sustain lactation significantly exacerbates sleep deprivation and parental exhaustion.

Additionally, parents often lack the necessary skills to facilitate the transition from bottle feeding to breastfeeding (29). They require professional guidance to assess appropriate timing (based on the infant’s condition and weight gain), adjust effective breastfeeding positions, and implement milk supplementation strategies. However, this skill deficit frequently prolongs and complicates the transition process, as parents vacillate between the “efficiency of bottle-feeding” and the “advantages of breastfeeding,” thereby increasing anxiety and impeding progress.

Furthermore, preterm infants often display subtle or ambiguous hunger and satiety cues (e.g., minimal movement, heart rate fluctuations), making it challenging for parents to distinguish between hunger, discomfort, and pain (25, 27). This can result in either overfeeding or underfeeding, which not only compromises nutritional intake and growth but also fosters parental self-doubt (feelings of inadequacy) and heightened sensitivity to external judgments regarding the infant’s growth (potential stigmatization). The confusion arising from unclear feeding signals further complicates the situation. The absence of targeted feeding support makes it difficult for parents to mitigate feeding-related stress, representing a significant barrier to family adaptation. For example:

*“I feel that my breastmilk is not enough. I can only express 60 mL at a time, and I feel bloated only when I do not express for half a day or even a day”* (21).

*“My baby breastfeeds frequently, and the doctor said that I need to pat the baby’s back after each feeding. However, I wonder if it’s because I’m doing it wrong and cannot get the air out of the baby’s stomach”* (24).

*“Satiation cues and cues to pause the feeding were also not well identified. One mother remarked, “When she first came home, she did not give you any of those signs; it was just sleeping”* (25).

*“I feel stressed when my infant cries, I do not know what he wants, whether he wants to suckle—but he has suckled.? I do not know why he cries so much”* (27).

*“… when I went home and tried baby on the breast the baby refused at all so like you would put the baby there and they lick the boobs and not suckle at all, so I did not breast feed because the baby was not cooperating so I went to therapies [at x hospital] they tried doing the oral massage but would not work”* (28).

*“My baby was admitted to the NICU because of premature birth, so he had been bottle-fed, and now my baby was discharged from the hospital, and my breast milk is also sufficient, but I find that the baby always cries when hungry, and not opening his mouth at all when he is full. How can I make my baby breastfeed”* (29).

#### 3.4.3 Meta-theme 3: continuity of services and resource requirements

##### 3.4.3.1 Sub-theme 7: accessibility of specialized resources

Parents of preterm infants frequently experience a strong need for professional support following hospital discharge, which often remains unmet due to systemic barriers in accessing resources, thereby exacerbating their negative experiences. When encountering abnormal symptoms (e.g., increased regurgitation, irregular breathing, unexplained crying), parents lack convenient and reliable access to professional counseling services (e.g., 24-h hotlines, accessible online platforms), and immediate consultation channels are frequently limited (18, 24, 29). Consequently, they may struggle in isolation or resort to unnecessary emergency department visits during late-night hours or crises, heightening their anxiety and potentially delaying the management of the preterm infant’s unstable condition.

Additionally, the home environment post-discharge differs substantially from the hospital setting, leaving parents in urgent need of professional home visits to assess environmental safety, provide guidance on practical care practices (e.g., feeding, monitoring), and correct errors in real time. The absence or inadequacy of home-visiting services compromises ongoing support (18, 29). Without such face-to-face, context-specific assistance, parental confusion and caregiving mistakes may persist, while fear and anxiety stemming from inexperience remain unresolved, further deepening parental exhaustion.

Moreover, insufficient pre-discharge training, lack of home environment assessments, poor integration with community resources, and inadequate preparation for transitioning from the highly supervised NICU environment to full caregiving autonomy reinforce feelings of helplessness in abnormal situations and concerns about growth, development, and long-term outcomes (24). Limited access to resources forces parents to make high-stakes decisions under stress, compounding the family burden with potential increases in healthcare costs. Frustration with caregiving may also lead to heightened self-doubt. For example:

*“Since the doctor in charge knows us better, it’s a good idea to have his contact information on the discharge record so that you can contact the doctor right away if you have questions”* (18).

*“Whenever we go to the community hospital for medical checkups, I hope the doctor will give us a lecture on feeding and caring for our premature babies and that he will come to our homes regularly to guide us in caring for our babies”* (18).

*“I seldom listen to my family’s advice and trust the guidance of healthcare professionals more. So, I hope the hospital can provide a platform, like WeChat or something similar, to facilitate our counseling”* (24).

*“If possible, a week before the baby is discharged from the hospital, I hope the hospital can arrange a comprehensive breastfeeding talk. Otherwise, the baby will really have nothing to do when he comes home!”* (24).

*“I hope that the NICU will set up a special hotline for us discharged preterm babies, so that when we encounter problems, we can call this special phone number and have special medical staff answer our questions and solve our problems!”* (29).

*“I hope that there will be professional pediatricians to conduct regular home visits for discharged preterm babies and provide different guidance for the babies at different stages of growth and development, in order to alleviate the difficulties and doubts of the families of our preterm babies in parenting”* (29).

##### 3.4.3.2 Sub-theme 8: optimization of health education formats

To effectively mitigate the fear and anxiety experienced by parents of preterm infants, as well as their sense of powerlessness stemming from insufficient nursing experience, the format of discharge preparation health education services must be urgently optimized (19, 22). The implementation of diversified educational approaches is imperative. Sole reliance on verbal explanations or printed materials proves inadequate in addressing the information-processing challenges faced by parents of preterm infants who are under significant stress and exhaustion (e.g., sleep deprivation, childcare fatigue). Multimodal interventions—including video demonstrations (e.g., proper feeding positions and cardiopulmonary resuscitation techniques), hands-on practice with manikins, illustrated digital brochures, and repeatable online courses—should be incorporated to improve comprehension of complex concepts (e.g., recognizing “preterm instability” and understanding developmental norms for corrected age). These methods enhance knowledge retention, reduce cognitive load, and strengthen parents’ capacity to respond to clinical abnormalities.

Reducing cognitive demands while increasing confidence in managing atypical situations is crucial. Enhancing paternal involvement represents a key strategy for alleviating maternal burden and improving family collaboration (18). This can be achieved by actively engaging fathers in skills training (e.g., bottle-feeding, bathing, first aid), customizing educational materials (such as step-by-step guides and checklists) to align with their learning preferences, and facilitating opportunities for joint practice. Such measures directly redistribute caregiving responsibilities—particularly feeding stress and nighttime care—thereby reducing maternal physical and emotional exhaustion. Concurrently, this approach enhances paternal role efficacy, diminishes skill-related alienation, and addresses concerns regarding growth, development, and long-term outcomes. Optimizing educational delivery and fostering paternal engagement are pivotal strategies for bridging information gaps, empowering parents, and counteracting their core adverse experiences. For example:

*“The hospital has a related education program, but I just have to travel. I do not have time to attend. New dads do not know anything, ah!”* (18).

*“He’s been in there so long, I do not know how he’s doing, and sometimes it’s not clear what he’s saying on the phone”* (19).

*“Health education should be organized in a variety of ways, both online and offline. If there is such a health education program, I will definitely participate in it, and many moms will too!”* (22).

##### 3.4.3.3 Sub-theme 9: integration of family and social resources

The need for continuous care for preterm infants after hospital discharge far exceeds the capacity of the average family, and the fragmentation and absence of key resources significantly exacerbate parental challenges. The first manifestation of this issue is the severe lack of human support for caregiving (18, 20, 23, 24). Primary caregivers, particularly mothers, often bear the burden of intensive 24-h care—including feeding, monitoring, and specialized care—without reliable assistance from family members or professional respite caregivers. This isolation traps mothers in an exhausting cycle of sleep deprivation and childcare, leaving them physically and mentally drained. The heavy family burden arises from their inability to attend to other responsibilities, while fathers may be compelled to work longer hours to meet financial needs, further straining family collaboration.

Secondly, the weak community healthcare collaboration system results in fragmented support (18, 19, 24). After discharge, there is inadequate information sharing and unclear responsibility allocation among family physicians, community nurses, rehabilitation organizations, and NICUs. Parents must repeatedly navigate between institutions to coordinate appointments, referrals, and ongoing medical care, such as developmental assessments, vaccinations, and counseling for abnormal conditions. This inefficiency increases the risk of missing critical information. Such siloed services not only deplete parents’ limited energy and delay problem resolution but also impose additional financial burdens through transportation costs, lost time, and potential duplicate testing expenses.

The failure to integrate resources forces parents to operate in isolation, often amid fear, anxiety, and helplessness. Any caregiving setbacks may be internalized as personal failures, heightening the risk of stigmatization. For example:

*“We would really like to have parents at home to help take care of the baby; it’s just too tiring for two people to take care of a baby! And since the parents are more experienced, I can focus more on my work!”* (18).

*“When the baby comes home, we hope that the medical staff will be the first to help in case of emergencies, and preferably someone will come to see us every now and then to guide us in our operations”* (18).

*“Because we are not doctors after all… subtle points are not clear”* (19).

*“During the time I was home, the baby was taken care of by my sister-in-law, who is a professional and has taken care of many premature babies”* (20).

*“My lover and I are both only children with no experience in raising children. We do not know anything—feeding, changing diapers, changing clothes, nothing. I feel that the baby will really suffer when they come home. I would love to have someone teach me or give me some advice”* (23).

*“I breastfed my first child without the help of my family and did not find breastfeeding difficult at the time. Now, this baby was born prematurely, with a very low birth weight, and I really could not have done it without family assistance!”* (24).

*“Is it okay to breastfeed while taking herbs? My friend said it’s okay after expressing about 20 mL of milk before breastfeeding, but I still do not feel comfortable without a clear answer from you and would like to know your advice!”* (24).

## 4 Discussion

### 4.1 Emphasizing the psychological preparedness of parents of preterm infants in the NICU and facilitating self-regulation of their roles

The birth of a preterm infant is a highly stressful event for families, presenting significant challenges. During preparation for hospital discharge, parents may experience complex emotions due to parent-infant separation, insufficient caregiving knowledge and skills, concerns about the child’s prognosis, and substantial financial burdens. These emotions can adversely affect parental mental health and may also negatively influence the growth and development of preterm infants (30). The findings of this study indicate that parents of preterm infants in the NICU frequently report pronounced negative physical and psychological experiences, with some also encountering role conflict, absence, and discomfort. These factors contribute to considerable challenges in preparing preterm infants for discharge. He et al. (31) further noted that parents of preterm infants express a need for psychological support from healthcare providers, family members, and society. Consequently, healthcare professionals should prioritize the psychological well-being of these parents. Interventions such as emotional support, informational support, narrative care, and positive thinking therapy may help alleviate negative emotions during the discharge preparation process. Additionally, enhancing communication between the multidisciplinary care team and parents can facilitate role adaptation and self-regulation, thereby improving discharge readiness (18, 19, 32).

### 4.2 Diversified health education for parents of preterm infants in the NICU to enhance their confidence in caregiving

The healthy development of preterm infants depends not only on medical technology but also on family support and care. Parents serve as the primary caregivers for preterm infants following hospital discharge (33). However, due to the closed management system of NICUs in China, parents have limited opportunities to interact with their preterm infants. The health education provided by medical staff to parents of preterm infants primarily occurs on the day of admission and discharge, with restricted communication and information exchange (34). Consequently, most parents lack adequate knowledge and skills to care for their preterm infants, experience low confidence in caregiving, and are unable to address the complex post-discharge care needs of preterm infants, thereby increasing the risk of readmission (5). This study found that most parents expressed a need for parenting knowledge and caregiving skills and desired hospitals to offer diverse health education programs. Although some hospitals in China have implemented family-integrated care (35), ternary integrated education (36), three-special care (37), and nursing empowerment programs (38), most NICUs continue to rely on traditional health education approaches due to constraints in human resources, medical resources, or institutional perceptions. Furthermore, the literacy levels and cultural backgrounds of preterm infants’ parents contribute to unsatisfactory educational outcomes (22).

Therefore, it is recommended that multidisciplinary health education be implemented and that a diversified health education system be developed to enhance educational content. For example, departments such as obstetrics, the NICU, and nutrition can collaborate to provide personalized health education tailored to the needs and characteristics of each family at different stages, with an emphasis on practical application. Additionally, with the rapid advancement of the internet, parents have access to diverse sources of information, yet many lack the ability to evaluate it effectively. This underscores the need for healthcare providers to emphasize internet-based health literacy education alongside professional health instruction, while also improving parents’ ability to critically assess health-related information. Finally, given the variability in preterm infants’ conditions and parental needs, the level of necessity and specific content of discharge preparation services will differ for each family. Consequently, future research should focus on developing a localized discharge preparation needs assessment tool. Based on the evaluation of need levels, targeted interventions—either standard or specialized discharge preparation services—can be provided accordingly.

### 4.3 Enhancing hospital-community-family support systems to improve the quality of post-discharge care for preterm infants

As the survival rate of preterm infants continues to improve, attention has shifted not only to their survival but increasingly to their quality of life (39). Therefore, ensuring a smooth transition from hospital to home support for preterm infants upon discharge eligibility is critically important. This study found that most parents desired healthcare professional support during home care for their preterm infants. Beck and Vo (40) demonstrated that reliable medical information support was positively correlated with parental confidence in post-hospital care for preterm infants. The United States has established a more comprehensive home care support system for preterm infants, significantly enhancing parental discharge readiness (41). However, China’s hospital-community-family transitional care remains underdeveloped, lacking an effective linkage mechanism (42), with room for improvement in continuity of care implementation. Consequently, medical institutions should develop an “Internet + hospital-community-family” support system. Hospitals could establish post-discharge continuity of care records for preterm infants through networked information platforms, effectively coordinating with community hospitals to provide integrated online and offline continuity of care support. Community staff should conduct regular follow-ups and provide home care guidance when necessary to enhance preterm infant home care quality.

## 5 Implications to practice

While experiencing negative emotions such as anxiety and fear, parents of preterm infants also demonstrate hope and a sense of responsibility regarding their infants’ discharge and recovery, reflecting the process of reconstructing their parental identity from passive recipients to active caregivers. This process highlights the importance of both standardized and individualized health education, as well as an external support system. Therefore, in future clinical practice, healthcare professionals should establish a dual-track emotional support mechanism that provides psychological guidance while reinforcing positive emotional support. Additionally, caregivers’ skills and decision-making abilities should be enhanced through simulation training, peer education, and similar interventions to foster self-efficacy. For example, parental confidence can be strengthened through the sharing of successful cases. Furthermore, pre-discharge health education should prioritize medical knowledge (e.g., respiratory management, feeding techniques) and emergency preparedness, supported by the development of tiered training programs and visual guidance tools. Finally, the synergistic effect of continuous medical team guidance, community resources, and policy support (e.g., extended parental leave) should be emphasized to establish a comprehensive hospital-community-family support network.

## 6 Limitations

This study has several limitations. First, the literature search was limited to Chinese and English sources, which may introduce language bias. Consequently, our findings may primarily reflect parental needs within the medical cultures of China and English-speaking countries, potentially failing to fully capture the unique experiences of parents of preterm infants in other cultural or linguistic contexts. Therefore, the generalizability of the results may be limited. Second, the search strategy was not formally validated for sensitivity using a predefined set of “sentinel studies.” Although we adapted search strategies from published high-quality systematic reviews in the field and further refined and expanded them, the absence of this objective validation step carries the risk of omitting relevant studies, potentially affecting the comprehensiveness of our findings. Additionally, 8 of the 12 included studies were authored by scholars from China. Due to variations in culture, healthcare policies, medical standards, and NICU management systems across countries and regions, the definition, implementation criteria, and core components of discharge preparation services may differ substantially. The unique management systems of NICUs in China (e.g., strict visitation policies, healthcare provider-led decision-making models, and uneven resource distribution) may particularly constrain the applicability of key elements such as discharge risk assessment tools, multidisciplinary team collaboration models, and intervention strategies. Furthermore, despite exhaustive efforts to obtain full-text articles through database searches, interlibrary loans, and direct correspondence with corresponding authors, we were unable to retrieve the full texts of potentially eligible studies. Their exclusion due to inaccessibility represents a potential source of bias in our review, as the findings and themes from these studies remain unknown. Finally, researchers hold diverse perspectives on and interests in discharge preparation services, while the psychological experiences of preterm infants’ parents regarding the need for such services are significantly influenced by regional cultural factors, potentially resulting in divergent findings.

## 7 Conclusion

This study integrated the experiences of parents of preterm infants in the NICU with discharge preparation services to examine parental needs and perceptions during discharge preparation. The synthesized findings indicate that parents of preterm infants in the NICU experienced a “three-dimensional imbalance” during discharge preparation, characterized by psychological isolation, information-skills anxiety, and discontinuity of care services. While these needs also occur among parents of full-term infants, they are significantly exacerbated by the heightened vulnerability and care complexity associated with preterm infants. Healthcare providers should prioritize psychological support, facilitate family role adaptation, provide tailored health education, and strengthen post-discharge support systems to enhance the caregiving capacity of parents of preterm infants.

## Data Availability

The original contributions presented in the study are included in the article/Supplementary material, further inquiries can be directed to the corresponding author.
