# Supplementary material for: Needs for discharge planning among parents of preterm infants in the NICU: a systematic review and meta-synthesis
Source: Front Public Health. 2025 Nov 6;13:1667721. doi: 10.3389/fpubh.2025.1667721 (PMC12631217; doi:10.3389/fpubh.2025.1667721)
Supplement: Supplementary file 1 [file Data_Sheet_1.docx]

**S1 File Database Retrieval Strategies**

****PubMed****

**#1：**infant, newborn[MeSH Terms]

#2：**(((((newborn*[Title/Abstract]) OR (neonate*[Title/Abstract])) OR (premature[Title/Abstract])) OR (infant, premature[Title/Abstract])) OR (infant[Title/Abstract])) OR (premature newborn[Title/Abstract])**

**#3：#1 OR #2**

**#4:** parents[MeSH Terms]

#5: **((((parent*[Title/Abstract]) OR (father*[Title/Abstract])) OR (mother*[Title/Abstract])) OR (maternal[Title/Abstract])) OR (paternal[Title/Abstract])**

**#6: #4 OR #5**

**#7:** **((((discharge planning[Title/Abstract]) OR (discharge preparation[Title/Abstract])) OR (discharge preparation service[Title/Abstract])) OR (plan for discharge[Title/Abstract])) OR (discharge readiness[Title/Abstract])**

**#8:** **qualitative research[MeSH Terms]**

**#9:** **((((((descriptive analysis*[Title/Abstract]) OR (qualitative study[Title/Abstract])) OR (phenomenology[Title/Abstract])) OR (grounded theory[Title/Abstract])) OR (thematic analysis*[Title/Abstract])) OR (content analysis*[Title/Abstract])) OR (interview[Title/Abstract])**

**#10: #8 OR #9**

**#11: #3 AND #6 AND #7 AND #10**

**the Cochrane Library**

#1:(Infant, Newborn OR Newborn* OR Neonate* OR premature OR infant, premature OR infant OR premature newborn):ti,ab,kw

#2:(parents OR parent* OR father* OR mother* OR maternal OR paternal):ti,ab,kw

#3:(Discharge Planning OR discharge preparation OR discharge preparation service OR plan for discharge OR discharge readiness):ti,ab,kw

#4:(Qualitative Research OR descriptive analy* OR qualitative study OR phenomenology OR grounded theory OR thematic analy* OR content analy* OR interview):ti,ab,kw

#5:#1 AND #12 AND #3 AND #4

**Embase**

#1:'infant, newborn':ab,ti OR newborn*:ab,ti OR neonate*:ab,ti OR premature:ab,ti OR 'infant, premature':ab,ti OR infant:ab,ti OR 'premature newborn':ab,ti

#2:parents:ab,ti OR parent*:ab,ti OR father*:ab,ti OR mother*:ab,ti OR maternal:ab,ti OR paternal:ab,ti

#3:'discharge planning':ab,ti OR 'discharge preparation':ab,ti OR 'discharge preparation service':ab,ti OR 'plan for discharge':ab,ti OR 'discharge readiness':ab,ti

#4:'qualitative research':ab,ti OR 'descriptive analy*':ab,ti OR 'qualitative study':ab,ti OR phenomenology:ab,ti OR 'grounded theory':ab,ti OR 'thematic analy*':ab,ti OR 'content analy*':ab,ti OR interview:ab,ti

#5:#1 AND #2 AND #3 AND #4

**MEDLINE**

#1：XB=( Infant, Newborn OR Newborn* OR Neonate* OR premature OR infant, premature OR infant OR premature newborn )

#2:XB=( parents OR parent* OR father* OR mother* OR maternal OR paternal )

#3:XB=( Discharge Planning OR discharge preparation OR discharge preparation service OR plan for discharge OR discharge readiness )

#4:XB=( Qualitative Research OR descriptive analy* OR qualitative study OR phenomenology OR grounded theory OR thematic analy* OR content analy* OR interview )

#5:#1 AND #2 AND #3 AND #4

**Web of Science**

**#1:TS=(****Infant, Newborn)**

**#2:AB=(Newborn* OR Neonate* OR premature OR infant, premature OR infant OR premature newborn)**

**#3:#1 OR #2**

**#4:TS=(****parents)**

**#5:AB=(parent* OR father* OR mother* OR maternal OR paternal)**

**#6:#4 OR #5**

**#7:AB=(****Discharge Planning OR discharge preparation OR discharge preparation service OR plan for discharge OR discharge readiness)**

**#8:TS=****（Qualitative Research)**

**#9: AB=(descriptive analy* OR qualitative study OR phenomenology OR grounded theory OR thematic analy* OR content analy* OR interview)**

**#10:#8 OR #9**

**#11:#3 AND #6 AND #7 AND #10**

****CNKI****

**#1:主题=早产儿**

**#2:篇关摘=新生儿(精确))OR未成熟儿(精确))OR患儿(精确)**

**#3:#1 OR #2**

**#4：主题=父母**

#5：**篇关摘=母亲(精确)OR父亲(精确)**

#6：#4 OR #5

#7：篇关摘=出院准备(精确) OR出院准备服务(精确)OR出院准备计划(精确)OR出院服务(精确)OR出院计划(精确)#8

#8：主题:质性研究

#9：篇关摘=定性研究(精确)OR扎根理论(精确)OR现象学(精确)OR人种学(精确)OR民族志(精确)OR叙事研究(精确)OR半构式访谈(精确)

#10:#8 OR #9

**#11:#3 AND #6 AND #7 AND #9**

**VIP**

#1:篇关摘=新生儿 OR 早产儿 OR 未成熟儿 OR 患儿

#2:篇关摘=父母 OR 母亲 OR 父亲

#3:篇关摘=出院准备 OR 出院准备服务 OR 出院准备计划 OR 出院服务 OR 出院计划

#4:篇关摘=质性研究 OR 定性研究 OR 扎根理论 OR 现象学 OR 人种学 OR 民族志 OR 叙事研究 OR 半结构式访谈

#5:#1 AND #2 AND #3 AND #4

**WANFANG**

#1:题目或关键词=新生儿 OR 早产儿 OR 未成熟儿 OR 患儿

#2:题目或关键词=父母 OR 母亲 OR 父亲

#3:题目或关键词=出院准备 OR 出院准备服务 OR 出院准备计划 OR 出院服务 OR 出院计划

#4:题目或关键词=质性研究 OR 定性研究 OR 扎根理论 OR 现象学 OR 人种学 OR 民族志 OR 叙事研究 OR 半结构式访谈

#5:#1 AND #2 AND #3 AND #4

**CBM**

#1:常用字段=新生儿 OR 早产儿 OR 未成熟儿 OR 患儿

#2:常用字段=父母 OR 母亲 OR 父亲

#3:常用字段=出院准备 OR 出院准备服务 OR 出院准备计划 OR 出院服务 OR 出院计划

#4:常用字段=质性研究 OR 定性研究 OR 扎根理论 OR 现象学 OR 人种学 OR 民族志 OR 叙事研究 OR 半结构式访谈

#5:#1 AND #2 AND #3 AND #4
