# Supplementary material for: Needs for discharge planning among parents of preterm infants in the NICU: a systematic review and meta-synthesis
Source: Front Public Health. 2025 Nov 6;13:1667721. doi: 10.3389/fpubh.2025.1667721 (PMC12631217; doi:10.3389/fpubh.2025.1667721)
Supplement: Supplementary file 2 [file Data_Sheet_2.docx]

**S2 File** **Data Extraction and Integration Process**

| **Descriptive themes** | **Sub-themes** | **Extracted results** | **Source** |
| --- | --- | --- | --- |
| Psychological and social support needs | Anxiety and fear. | Health Uncertainty | *"I'm wondering if she'll fall behind other kids her age and if there are any other implications?" [19]*  *"He sleeps in a strange position, always with his head to the side and his body in an S-shape. I don't know if it will cause curvature of the spine or something." [*22*]*  *"The time since the baby was discharged from the hospital... I'm worried about the change in his condition..." [*23*]*  *"I keep checking her breathing because I'm afraid she's going to stop" [27]* |
|  |  | Care Confidence Deficit | *"When I was discharged from the hospital, I thought it would be good to come back, but I didn't realize that it would be totally different after I came back! My baby was not feeding well and was often bloated, so I was very anxious and felt that it was better to stay in the hospital." [*18*]*  *"I didn't dare to hold her for the first two days after she came back, thinking she was too small and soft. I didn't dare to walk after picking her up for fear that she would fall." [*22*]*  *"I didn't know how to do anything; it was my grandmother who did it, and I didn't dare to take him out of the hospital without her help. One day when Grandma went out, the baby's poop got everywhere, and I was so anxious that I cried along with him, calling my mom to tell her to come back soon." [*23*]*  *"You don’t know what you are feeling at that point.It was the… okay, the thing is, it comes with fear,you are excited, you really want to go home, but this child is still tiny, because their discharge weight is 1.75 (Kg) up to 2kgs depending on if the baby is feeling good. So, if the baby is stable… mine was born at 1700 grams. What are you going to do with this baby? You know, here you were confident, if he vomits a little, “doctor the baby is vomiting”, he tells you, “aahh… mum bend the baby and do this”. Now you’ve been told to go home, you will be alone, whatwill happen? "**[*28*]* |
|  | Family role adaptation | Conflicting Parenting Experiences | *"It is said that the eldest is raised according to books and the youngest according to pigs. In our case, the oldest was raised according to books, and the second had to be raised according to books even more. Because she was born prematurely, I feel that she is completely different from the oldest when she was a baby, so I can't raise her with the same experience as the oldest. I feel like I'm learning all over again!" [*22*]* |
|  |  | Caregiver Collaboration | *"I've been studying lately... I hope I can be a competent husband and father." [*18*]*  *"...I work during the day and take care of them at night after work..." [*19*]*  *"I was particularly challenged in taking care of my infant. It was almost the same as infants in general. But I had to undertake more intensive monitoring of my infant." [*27*]*  *"I am the father of a baby, and I am always criticized by my family for being clumsy and not being allowed to touch the baby. I want to take care of my child, and I hope my family recognizes and supports me." [29]* |
|  |  | Financial Stresses | *"He lives inside, we live outside. It costs money. It's a big burden." [*19*]* |
|  | Peer and emotional support | Experience Sharing | *"Many mothers of preterm babies shared their breastfeeding experiences in the WeChat group, and I would ask them questions when I encountered problems. From these interactions, I gained a lot of breastfeeding skills that I couldn't learn from books!" [*24*]*  *"I think it's very good that the hospital has set up a group for mothers of preterm babies. For example, some babies will have hemolysis after birth, and to a certain extent, they will need to have their blood replaced. Mothers who don't know the situation will be scared to death when they hear the term "blood exchange," but it's different when we communicate with each other in the group. I feel much more relieved when I learn from other people's experiences, and I usually consult with the group when I have questions about breastfeeding. Everyone is very willing to help!" [*24*]*  *"We would burp our babies after feeding, but we could never burp them well, so we joined a couple of NICU preemie family parents' WeChat groups. Experienced parents in the group taught us. It's good to be in this group. When we feel bad, parents who have had similar experiences share their own stories with us to relieve our stress and help us get through this difficult time!" [29]* |
|  |  | Emotional Catharsis | *"I used to cry in secret when my family wasn't around. My lover and friends have been doing the thinking for me, and I feel better now." [*24*]*  *"After the baby came home, the color of the stool got lighter and lighter. Suspecting it was biliary atresia, I became very depressed. I didn't dare to talk to the older people at home, afraid they would feel the pressure. Sometimes I would talk to my friends, and they would comfort me, which made the pressure feel less." [29]* |
| Information and skills support needs | Medical knowledge guide | Feeding Issues | *"Baby's stools often have some white stuff mixed in... Is it a digestive problem?" [*19*]*  *"I heard that rice and soybean paste are highly nutritious, but I was worried about the baby's indigestion, so I didn't dare to feed it. Now I'm really scared to think about it—if it causes indigestion, I'll have to be hospitalized again." [*21*]*  *"Due to the fear that babies do not adapt to breastfeeding... But the baby didn't want to eat it, so I added some formula milk." [*21*]*  *"The baby spits up a lot; twice it came out of his nose, and the family had to take turns sleeping at night. How scary if the spitting up goes unnoticed!" [23]*  *"When the baby was first discharged from the hospital, she cried very hard and would not breastfeed. I thought she was sick, but the doctor checked her and said she was fine. I think I'm too sensitive and get very nervous when she doesn't want to breastfeed, and I feel really tired of this!" [*24*]*  *"My baby was born prematurely and admitted to the NICU. Although I was told to express or pump breastmilk during the hospitalization, I still didn’t know how to breastfeed after my baby was discharged" [29]* |
|  |  | Vital Signs Monitoring | *"The doctor said to monitor the baby's temperature after discharge. How should I measure it? Which part of the body should be measured? For how long?" [20]*  *"Baby has been sleeping most of the day since coming home ... Is this normal?" [*20*]*  *"Why is my child breathing so fast—much faster than me? Is he having trouble breathing? The night I was discharged from the hospital, I took him back because it seemed like he was breathing quickly. Although the doctor said he was fine, I still felt uneasy." [*21*]*  *"I've been worried about my baby's belly button rising 2 centimeters when he cries." [*22*]* |
|  |  | Developmental Assessment | *"Because it is a premature baby... It may have growth retardation after discharge from the hospital" [*19*]*  *"Id about his development. Since he was born prematurely, he's different from normal babies." [*22*]*  *"I'm worried about his development. Look at me now; I always think he's short." [*22*]*  *"Like I have a neighbor—they are one month apart from my son. But you know, my son can’t walk on his own, but that kid can walk now... Yeah. So, someone goes, ‘Ah, when was your kid born?’ ‘You know, um, has he not started walking..." [*28*]* |
|  | Practical skills training | Primary Care | *"Taking a shower sounds easy, but it's hard to do... I wish someone were there to guide me." [*20*]*  *"There are so many straps on the baby's clothes, I really don't know which one to tie to which one, and the bag I wrapped myself in was loosened by the baby's kick!" [20]*  *"When I was first discharged from the hospital, I didn't know how to hold him, I didn't know how to wrap him, and I didn't know how to burp him because his head kept swaying back and forth when he sat up." [*22*]*  *"Sometimes babies sleep for 6–7 hours without waking up, and I want to wake them to feed them, but I can't get them to wake up. I'm confused." [23]* |
|  |  | Emergency Management | *"When I first came home from the hospital, I was worried that he had stopped breathing. He stopped breathing while breastfeeding; he couldn't breathe enough and turned purple. I was very worried." [*22*]*  *"Just got back from the hospital... and then her limbs were shaking." [*22*]*  *"He was hypothermic, so I was a little nervous and upset... So of course I got frustrated and started crying." [*26*]* |
|  |  | Specialty Care | *"The baby's mouth is so small, I don't dare to open it... I'm really at a loss when it comes to giving medicine to my baby" [*22*]*  *"When will the jaundice on the baby's face go away completely? Is there a problem? Should I go to the hospital?" [23]*  *"Why is the umbilical cord not falling off after one month, and when will it fall off? I'm afraid to take a bath until the umbilical cord falls off." [23]*  *"I did KMC for three days after returning home from the hospital, but I didn't it anymore because my infant was healthy. KMC was difficult and I needed to be helped by others as I cannot do it alone. " [*27*]*  *"Yeah, they explained to us how to handle the baby well at home, especially insisting that we continue with kangaroo mother care... Until they reach those kilograms. Kangaroo mother care helps the baby gain weight fast..." [*28*]* |
|  | Specialized support for feeding | Breastfeeding Disorders | *"Breast milk is very thin, like water. Is this breast milk not nutritious? Babies get hungry in one to two hours, and 50 ml of breast milk can only last one hour, while formula feeding can be given at intervals of three to four hours." [21]*  *"I feel that my breastmilk is not enough. I can only express 60 ml at a time, and I feel bloated only when I don't express for half a day or even a day." [21]*  *"My baby breastfeeds frequently, and the doctor said that I need to pat the baby's back after each feeding. However, I wonder if it's because I'm doing it wrong and can't get the air out of the baby's stomach." [*24*]*  *"The baby was too weak to suckle milk... eventually, it gave up." [*24*]*  *"… when I went home and tried baby on the breast the baby refused at all so like you would put the baby there and they lick the boobs and not suckle at all, so I didn’t breast feed because the baby was not cooperating so I went to therapies [at x hospital] they tried doing the oral massage but would not work" [*28*]* |
|  |  | Bottle-to-breast transition techniques | *"My baby was admitted to the NICU because of premature birth, so he had been bottle-fed, and now my baby was discharged from the hospital, and my breast milk is also sufficient, but I find that the baby always cries when hungry, and not opening his mouth at all when he is full. How can I make my baby breastfeed" [*29*]* |
|  |  | Feeding Signal Recognition | *"...This time she went for 3 hours, and then the next time she didn't eat for 4 hours. I thought I was late to the party, and I was late to feed her..." [*25*]*  *"Satiation cues and cues to pause the feeding were also not well identified. One mother remarked, “When she first came home, she didn’t give you any of those signs; it was just sleeping." [*25*]*  *"I feel stressed when my infant cries, I do not know what he wants, whether he wants to suckle—but he has suckled.? I don't know why he cries so much" [*27*]* |
| Continuity of services and resource requirements | Accessibility of specialized resources | Immediate Counseling Channels | *"Since the doctor in charge knows us better, it's a good idea to have his contact information on the discharge record so that you can contact the doctor right away if you have questions." [*18*]*  *"I seldom listen to my family's advice and trust the guidance of healthcare professionals more. So, I hope the hospital can provide a platform, like WeChat or something similar, to facilitate our counseling." [24]*  *"I hope that the NICU will set up a special hotline for us discharged preterm babies, so that when we encounter problems, we can call this special phone number and have special medical staff answer our questions and solve our problems!" [29]* |
|  |  | Home Visiting Services | *"Whenever we go to the community hospital for medical checkups, I hope the doctor will give us a lecture on feeding and caring for our premature babies and that he will come to our homes regularly to guide us in caring for our babies." [*18*]*  *"I hope that there will be professional pediatricians to conduct regular home visits for discharged preterm babies and provide different guidance for the babies at different stages of growth and development, in order to alleviate the difficulties and doubts of the families of our preterm babies in parenting." [29]* |
|  |  | Transition Preparation | *"If possible, a week before the baby is discharged from the hospital, I hope the hospital can arrange a comprehensive breastfeeding talk. Otherwise, the baby will really have nothing to do when he comes home!" [*24*]* |
|  | Optimization of health education formats | Diversity Missions | *"He's been in there so long, I don't know how he's doing, and sometimes it's not clear what he's saying on the phone" [*19*]*  *"Nowadays, the Internet is particularly convenient. You can also send some videos in the public number for everyone to learn." [*22*]*  *"Health education should be organized in a variety of ways, both online and offline. If there is such a health education program, I will definitely participate in it, and many moms will too!" [*22*]* |
|  |  | Father Involvement Support | *"What to buy... I don't know. My wife prepares it all." [*18*]*  *"The hospital has a related education program, but I just have to travel. I do not have time to attend. New dads do not know anything, ah!" [18]* |
|  | Integration of family and social resources | Caregiving Workforce Support | *"We are in great need of family help! It's best to have elders help with the baby, and it's even better if you can hire a sister-in-law! They're more experienced, so I'm relieved!" [*18*]*  *"We would really like to have parents at home to help take care of the baby; it's just too tiring for two people to take care of a baby! And since the parents are more experienced, I can focus more on my work!" [*18*]*  *"During the time I was home, the baby was taken care of by my sister-in-law, who is a professional and has taken care of many premature babies." [20]*  *"My lover and I are both only children with no experience in raising children. We don’t know anything—feeding, changing diapers, changing clothes, nothing. I feel that the baby will really suffer when they come home. I would love to have someone teach me or give me some advice." [*23*]*  *"I breastfed my first child without the help of my family and didn't find breastfeeding difficult at the time. Now, this baby was born prematurely, with a very low birth weight, and I really couldn't have done it without family assistance!" [*24*]* |
|  |  | Community Health Collaborative | *"When the baby comes home, we hope that the medical staff will be the first to help in case of emergencies, and preferably someone will come to see us every now and then to guide us in our operations." [*18*]*  *"Because we're not doctors after all... subtle points are not clear." [*19*]*  *"Is it okay to breastfeed while taking herbs? My friend said it's okay after expressing about 20 mL of milk before breastfeeding, but I still don't feel comfortable without a clear answer from you and would like to know your advice!" [*24*]* |
